# Utilization of diabetes medication and cost of testing supplies in Saskatchewan, 2001

**DOI:** 10.1186/1472-6963-6-159

**Published:** 2006-12-12

**Authors:** Jeffrey A Johnson, Sheri L Pohar, Kristina Secnik, Nicole Yurgin, Zeenat Hirji

**Affiliations:** 1 Institute of Health Economics, Edmonton, Canada; 2 School of Public Health, University of Alberta, Edmonton, Canada; 3 Eli Lilly, Indianapolis, USA; 4 Eli Lilly Canada, Toronto, Canada

## Abstract

The purpose of this study was to describe the patterns of antidiabetic medication use and the cost of testing supplies in Canada using information collected by Saskatchewan's Drug Plan (DP) in 2001. The diabetes cohort (n = 41,630) included individuals who met the National Diabetes Surveillance System (NDSS) case definition. An algorithm was then used to identify subjects as having type 1 or type 2 diabetes. Among those identified as having type 2 diabetes (n = 37,625), 38% did not have records for antidiabetic medication in 2001. One-third of patients with type 2 diabetes received monotherapy. Metformin, alone or in combination with other medications, was the most commonly prescribed antidiabetic medication. Just over one-half of the all patients with diabetes had a DP records for diabetes testing supplies. For individuals (n = 4,005) with type 1 diabetes, 79% had a DP record for supplies, with an average annual cost of $472 ± $560. For type 2 diabetes, 50% had records for testing supplies, with an average annual cost of $122 ± $233. Those individuals with type 2 diabetes who used insulin had higher testing supply costs than those on oral antidiabetic medication alone ($359 vs $131; p < 0.001).

## Background

Diabetes affects approximately 5% of all Canadians aged 20 years or older, with the prevalence rising with age [1] Type 2 diabetes accounts for 90% of the diagnosed cases of diabetes in Canada, while approximately 10% of diabetes cases are attributed to type 1 diabetes [1]. The National Diabetes Surveillance System (NDSS) monitors epidemiologic trends in diabetes in Canada and recently reported on the prevalence of the disease and mortality in individuals with diabetes [1]. It was estimated that approximately 5.2% of the non-First Nations Saskatchewan population over the age of 20 had diabetes (4.9% of females and 5.5% of males) [1]. Both type 1 and type 2 diabetes can be associated with a substantial burden for individuals with the disease, their families and society as a whole.

The total costs of diabetes to health care systems have been previously estimated for US and Canada [2,3] These previous estimates have been modeled using data from various sources. In order to better understand the magnitude of health care costs associated with the management of diabetes, administrative health care data from Saskatchewan have been used previously to estimate health care costs of a cohort of 45,716 individuals identified as having diabetes during 1991 to 1996 [4,5]. Costs for hospitalizations and day surgeries accounted for over 60% of health care expenditures, with prescription expenditures accounting for 20%, physician services accounting for 16%, and dialysis services accounting for 4% [4]. A large proportion of health care expenditures for people with diabetes is for macrovascular comorbidity [5]. We also noted that in 1996, diabetes testing supplies accounted for approximately 13% of the overall prescription expenditures for people with diabetes [6].

Antidiabetic medications and diabetes testing supplies play an important part in the management of diabetes, but are associated with substantial and increasing costs for provincial health care programs [4,5,7]. The current clinical practice guidelines for the management of diabetes in Canada were released in 2003 [8]. The guidelines provide recommendations for self-monitoring of blood glucose, and call for aggressive management (e.g., earlier initiation of therapy) to avoid complications of diabetes. Despite these guidelines, treatment gaps exist, whereby the actual patterns of practice do not meet clinical practice guideline recommendations. Treatment gaps for glycemic control have been reported in Canada, although the patterns of practice are often described for selected samples [9-11]. Harris' recent report on patterns of glycemic control in Canada reflected practice patterns in a sample of patients with type 2 diabetes from the primary care setting [10]; although it included patients from across the county, it was based on a convenience sample and so may not be reflective of the full population.

The purpose of this population-based study was to describe the utilization of antidiabetic medications and diabetes testing supplies within a provincial drug program for individuals with diabetes. We were particularly interested in the population of type 2 diabetes and their patterns of antidiabetic medication use and associated costs of diabetes testing supplies during 2001.

## Methods

### Data sources and study population

Administrative databases from Saskatchewan Health that contain information on prescription drug use, hospitalizations, and physician services for all eligible residents of Saskatchewan were used in the analyses [12]. More than 99% of the province's population is covered by Saskatchewan Health. Beneficiaries whose prescription drug benefits are provided by a federal government agency (e.g., First Nations & Inuit Health, Health Canada) are not eligible for Saskatchewan Health's prescription drug benefits and therefore their prescriptions are not captured in the prescription drug database. With these exclusions, approximately 90% of the covered population are eligible for prescription drug benefits [12].

Saskatchewan Health's administrative databases from 1991 to 2001 were used to identify non-registered Indian prevalent and incident diabetes cases in the 2001 calendar year. Using the NDSS criteria, [1] individuals were considered to have diabetes if they had two physician visits with a diagnosis of diabetes (ICD-9 code of 250) on two different days within any contiguous 730-day period or one hospitalization with a discharge diagnosis of diabetes (ICD-9 code of 250 from the first three diagnostic fields) during the 11-year period [1]. The NDSS case definition is typically applied for individuals 20 years and older; for this report we have also included those less than 20 years of age. From 1991 to 2001, 57,774 non-registered Indians were identified as having diabetes. The 2001 cohort included 41,630 diabetes cases (3108 incident and 38,522 prevalent cases). Approximately 16,144 diabetes cases had exited the study by 2001 either due to death or termination of Saskatchewan Health coverage.

Individuals were then categorized as type 1 or type 2 diabetes based on patterns of drug use. Individuals who were on insulin only in 2001 and had no history of oral medication use were classified as having type 1 diabetes; all others were considered to have type 2 diabetes.

The compiled study dataset included all prescriptions for antidiabetic medications drugs and diabetes testing supplies covered by the DP for all subjects identified with diabetes. The category for diabetes-related testing supplies included in the data sets did not separate blood glucose test strips and urine ketone testing agents; however, we expect the majority of records are for blood glucose test strips. Blood glucose meters, lancets, swabs, etc., were not included in the diabetes-related testing supplies category. Expenditures for diabetes testing supplies were compiled from this administrative database. This dataset contains the total approved and government portion of the cost. This analysis was restricted to utilization during 2001 and costs are therefore reported as 2001 Canadian dollars. The research was approved by the Health Research Ethics Board of the University of Alberta.

### Data analysis

Descriptive statistics were used to characterize the cohorts and patterns of antidiabetic medication and diabetes testing supplies utilization for the 2001 calendar year. Total costs of diabetes testing supplies were compared for type 1 and type 2 diabetes and among type 2 diabetes subjects in four treatment groups, defined by DP records for prescription medications: no DP records for any diabetes-related medication, oral antidiabetic medication alone, insulin alone or insulin in combination with oral antidiabetic medications. As a proxy for disease duration in 2001, subjects were also categorized according to quartiles of duration of follow-up between 1991 and 2001 based on when they were identified as having diabetes: less than 3.0 years, 3.0 to 5.9 years, 6.0 to 9.9 years and 10.0 years or greater. Patterns of medication use and total costs of diabetes testing supplies were then compared between type 1 and type 2 diabetes for each quartile of follow-up and across quartiles within each type of diabetes. The nonparametric Kruskal-Wallis test was used for all comparisons.

## Results

We identified 41,630 individuals with diabetes who were eligible for prescription benefits and had not exited the study due to death or coverage termination by 2001. The majority (n = 37,625; 90%) of these were categorized as having type 2 diabetes (Table 1). As would be expected, subjects with type 2 diabetes were older than those with type 1 diabetes (70.7 ± 16.2) versus 58.5 ± 22.1, p < 0.001). Subjects with type 1 and type 2 diabetes also differed with respect to duration of follow-up and location of residence (Table 1).

**Figure 1:**
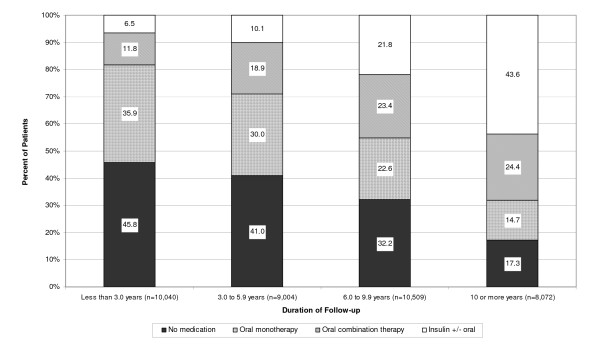
Antidiabetic Medication use in Type 2 diabetes in 2001 by Duration of Follow-up.

Among those identified as having type 2 diabetes, approximately one-third of patients received monotherapy. Metformin was the most commonly prescribed antidiabetic drug, with almost one-quarter of pharmacologically treated subjects receiving metformin monotherapy in 2001, and approximately 65% receiving metformin, alone or in combination with other medications (Table 2). Approximately 12% of all individuals with type 2 diabetes received insulin in 2001 (Table 2). Over one-third of subjects with type 2 diabetes (n = 14,157; 38%) did not have records for antidiabetic medications in 2001, although this varied substantially by duration of diabetes follow-up (Figure 1). A clear pattern of increasing medication use with longer duration of follow-up was apparent (Figure 1), with approximately 44% of individuals with 10 or more years of follow-up receiving insulin therapy.

Just over one-half (53%) of the all patients with diabetes had a DP record for diabetes testing supplies (Table 3). For those individuals with type 1 diabetes, 79% (n = 3,143) had an DP record for diabetes testing supplies. These individuals had a mean (standard deviation [SD]) of 5.8 (6.4) records in 2001, with a mean (SD) annual total cost of $472 ($560). For all subjects identified with type 2 diabetes, 50% (18,771) had records for testing supplies (Table 3), with an overall mean (SD) of $122 ($233). When considering the various therapies for individuals with type 2 diabetes, those managed with no antidiabetic medication had the lowest costs of testing supplies (Table 3). Those individuals with type 2 diabetes using insulin, alone or in combination with oral medication, had higher testing supply costs than those on oral antidiabetic medication ($359 vs $131; p < 0.001).

Duration of follow-up in this sample was associated with cost of diabetes testing supplies (Table 4). Interestingly, however, the association was in different directions for type 1 and type 2 diabetes. One-half of individuals identified with type 1 diabetes had follow-up for at least 10 years, but had the lowest cost of testing supplies during 2001 at $464, compared to $575 for those with type 1 diabetes for 3 years or less (Table 4). The cost of testing supplies increased with duration in those identified with type 2 diabetes, from $107 for the shortest duration to $181 for those with type 2 diabetes for 10 years or more (Table 3).

## Discussion

Our observations indicate that metformin was the most commonly used antidiabetic medication in Saskatchewan in 2001, used alone or in combination by two-thirds of patients with type 2 diabetes who were treated with medication. This pattern is consistent with the current CDA guidelines, which recommend it as first line therapy for most patients with diabetes [8]. Interestingly, however, over one-third of patients with type 2 diabetes were not dispensed antidiabetic medication in 2001, and were presumably managed by lifestyle (i.e., diet and exercise) alone. While this may seem high at first glance, it is, in fact, similar to estimates for other countries [13-15]. On the other hand, Harris et al., in the recently reported Diabetes in Canada Evaluation (DICE) Study, only 15% of patients with type 2 diabetes were managed by lifestyle alone, although this varied widely depending on the duration of diabetes [10]. In our data, 46% of individuals with type 2 diabetes had no records for drug therapies made during the first 3 years after identification of their diabetes.

We were limited in this analysis to administrative records, making it difficult to determine the appropriateness of the observed patterns of medication use relative to actual glycemic control or other clinical characteristics (e.g., BMI). A number of previous reports in Canada and elsewhere have indicated that treatment gaps exist, with suboptimal management of glycemic control in people with type 2 diabetes [9-11,16]. We suspect that our population would have similar characteristics to those previous reports of Canadian populations. For example, Harris et al., in the recent DICE Study, found that while the mean A1c was 7.3%, about one-half of subjects with type 2 diabetes were not at target (i.e., A1c < 7.0%) [10]. Similarly, Toth et al. found that the average A1c among a sample of people with type 2 diabetes in northern Alberta was 7.3%, but 50% of subjects were not at target [9]. Furthermore, the majority of subjects not at target A1c were on single or no antidiabetic medication [9].

The current clinical practice guidelines for diabetes management in Canada call for more aggressive management for people with diabetes, including glycemic control [8]. It should be noted that all of the previous evaluations were undertaken prior to the wide dissemination of the recent CPG, which were released in December 2003. Given the typically slow rate of adoption of new evidence into practice, we suspect that patterns of practice have improved since 2001, but not substantially so. The discordance between recommendations and actual practice can be partially attributed to "clinical inertia," which has been defined as the recognition of a problem with a patient's management but a failure to act [16]. Shah et al. recently showed that clinical inertia is common for diabetes, regardless of whether a diabetes specialist has been involved in patient care[16]. Perhaps even more concerning, given that macrovascular disease is the leading cause of death in this population, is the evidence of even greater treatment gaps for management of cardiovascular risk [17,18].

We also highlight the costs associated with diabetes self-testing in Saskatchewan in 2001. As would be expected, individuals with type 1 diabetes, followed by those with type 2 diabetes on insulin, had the highest expenditures for diabetes testing supplies. Interestingly, however, overall only one-half of subjects identified with diabetes had records for diabetes testing supplies. This was an increase over our previous report, where only 43% of diabetic subjects in Saskatchewan in 1996 had DP records for testing supplies [6]. The increase appears to be primarily in subjects with type 2 diabetes managed with oral medications, from 49% in 1996 to 63% in 2001 [6].

Self-monitoring of blood glucose (SMBG) is considered a cornerstone of self-management for all people with diabetes [8,19]. There are, however, no specific guidelines on testing frequency, particularly for people with type 2 diabetes. Indeed, the precise role and benefits of SMBG in patients with type 2 diabetes is unclear. While there appears to be some benefit of testing for those on insulin to avoid hypoglycemia [20], the little evidence associating SMBG with better glycemic control in patients not on insulin is weak and conflicting [20-23]. The lack of good evidence has prompted some opinion leaders to suggest that SMBG for people with type 2 diabetes not receiving insulin is a "waste of money"[24]. Two-thirds of the total cost of diabetes testing supplies in Saskatchewan in 2001 were incurred for individuals with type 2 diabetes, and approximately 80% of the individuals with type 2 diabetes who had records for diabetes testing supplies were managed by lifestyle alone or were on oral medications only. As provincial health systems provide insurance coverage for diabetes testing supplies, studies into the clinical and economic impact of this policy should be considered.

Our estimates for drug and testing supply use should be considered in light of several limitations. First, while there is good evidence of validity of the NDSS criteria for identifying diabetes cases, some cases may still be missed [25]. Individuals whose physicians do not use fee-for-service billing, for example, may not meet the NDSS criteria for diabetes unless they are hospitalized for diabetes. In Saskatchewan's northern health districts, some physicians were under salary during the study period and did not bill for their services under the fee-for-service structure [4]. Furthermore, the NDSS criteria have only been validated in adults over the age of 20; we applied the same criteria to identify diabetes in all age groups. The result of both of these limitations is an underestimation of the prevalence of diabetes, and therefore utilization and cost of services. In addition, our approach to identifying individuals with type 1 or type 2 diabetes has not been validated against chart audits. Nonetheless, by considering the patterns of medication use, we identified the majority of people (90%) as having type 2 diabetes, which is what would be expected with the known epidemiology of diabetes [1]. Finally, our utilization and cost estimates are derived from 2001 data, and may not reflect more contemporary patterns of utilization and cost.

## Conclusion

In Saskatchewan in 2001, metformin, alone or in combination, was the most frequently dispensed oral antidiabetic medication. A longer history of diabetes was associated with increased use of oral medications and insulin therapy. Insulin was used in approximately 12% of patients with type 2 diabetes and was associated with approximately three times higher expenditure on diabetes testing supplies compared to patients on oral anti-diabetic medications. Individuals with type 1 diabetes had the highest expenditures for testing supplies.

## Authors' contributions

JAJ, KS, NY and ZH conceived of the study concept. JAJ coordinated the  project, acquired the data, and prepared the initial draft of the manuscript. SP conducted the data analysis and critically revised the  manuscript.  KS, NY and ZH also participated in planning the analysis and critically revised the manuscript. All authors read and approved the final manuscript.

## Pre-publication history

The pre-publication history for this paper can be accessed here:



## Figures and Tables

**Table 1:** Diabetes Cohorts, Saskatchewan 2001

	**Type 1**	**Type 2**
N (%)	4,005 (9.6%)	37,625 (90.4%)
Age*** – mean (SD), years	58.5 (22.1)	70.7 (16.2)
Sex – N (%), males	2,168 (54.1)	19,963 (53.1)
Residence*** – N (%), rural	1,734 (43.3)	17,469 (46.4)
Duration of follow-up*** – N (%)		
Less than 3 years	421 (10.5)	10,040 (26.7)
3.0 to 6.0 years	458 (11.4)	9,004 (23.9)
6.0 to 10.0 years	1,115 (27.8)	10,509 (27.9)
10.0 or more years	2,011 (50.2)	8,072 (21.5)

*** p < 0.001

**Table 2:** Utilization of Diabetes Medication for Type 2 diabetes, Saskatchewan 2001

**Therapy**	**Number of Patients with Type 2 Diabetes**	**% of Total Patients with Type 2 Diabetes (n = 37625)**	**% of Type 2 Diabetes Patients Receiving Antidiabetic Therapy (n = 23,468)**
**Monotherapy**	**12814**	**34.1**	**54.6**
SU	4803	12.8	20.5
Met	5767	15.3	24.6
TZD	32	0.1	0.1
Insulin	2212	5.9	9.4

**Oral Combination Therapy**	**7493**	**19.9**	**31.9**
SFU+Met	6764	18.0	28.8
SFU+TZD	59	0.2	0.3
Met+TZD	137	0.4	0.6
SFU+Met+TZD	533	1.4	2.3

**Oral + Insulin Combination**	**2151**	**5.7**	**9.2**
SFU+Ins	142	0.4	0.6
Met+Ins	1202	3.2	5.1
TZD+Ins	53	0.1	0.2
SFU+Met+Ins	554	1.5	2.4
SFU+TZD+Ins	15	0.0	0.1
Met+TZD+Ins	88	0.2	0.4
SFU+Met+TZD+Ins	97	0.3	0.4

**Other Antidiabetic Drugs**	**1010**	**2.7**	4.3

**No diabetes drugs**	**14157**	**37.6**	-

**Total**	37625	100	100

**Table 3:** Utilization and cost of diabetes testing supplies by Type of Diabetes and Treatment, Saskatchewan 2001

	**Number of Cases**	**Number (%) of Subjects with ≥ 1 record for testing supplies**	**Average (SD) Number of Records**	**Average (SD) DP share (Can$)**	**Average (SD) Patient Share (Can$)**	**Average (SD)^**A, B **^Total (Can$)**
**Type 1**	4005	3143 (78.5)	5.8 (6.4)	231.71 (408.53)^A^	240.60 (343.81)^A^	472.31 (560.75)^A^
(% of Total)	(9.6%)			(49.1%)	(50.9%)	
**Type 2**						
No medication	14157	3169 (22.4%)	0.5 (1.5)	11.78 (62.14)^B^	23.69 (72.61)^B^	35.47 (107.22)^B^
(% of Type 2)	(37.6%)			(33.2%)	(66.8%)	
Oral anti-diabetic agent only	18910	11814 (62.5)	2.0 (2.6)	57.78 (132.42)	73.42 (110.65)	131.21 (189.78)
(% of Type 2)	(50.3%)			(44.0%)	(56.0%)	
Insulin only	2212	1751 (79.2)	4.6 (5.0)	194.53 (296.40)	153.33 (233.26)	347.86 (409.39)
(% of Type 2)	(5.9%)			(55.9%)	(44.1%)	
Insulin and oral anti-diabetic agent	2346	2037 (86.8)	4.9 (4.4)	220.08 (286.55)	148.48 (187.50)	368.56 (362.67)
(% of Type 2)	(6.2%)			(59.7%)	(40.3%)	
Type 2 Total	37625	18771 (49.9)	1.8 (2.9)	58.63 (155.18)^A^	64.09 (122.86)^A^	122.72 (233.88)^A^
(% of Total)	(90.4%)			(47.8%)	(52.2%)	

A p < 0.001 between type 1 and type 2 diabetes using the Kruskal Wallis Test

B p < 0.001 between treatment groups within type 2 diabetes (i.e. no medication, oral anti-diabetic agent only, insulin only, insulin and oral anti-diabetic agent) using the Kruskal Wallis Test

**Table 4:** Utilization and cost of diabetes testing supplies by type of diabetes and duration, Saskatchewan 2001

	**Number of Cases**	**Number (%) with ≥ 1 record for testing supplies**	**Average (SD) Number of Records**	**Average (SD) Total (Can$)**
**Type 1**				
Less than 3.0 years	421	358 (85.0)	6.8 (6.8)^A, C^	574.51 (583.95)^A, C^
(% of Type 1)	(10.5)			
3.0 to 5.9 years	458	355 (77.5)	6.1 (6.8)	492.00 (552.76)
(% of Type 1)	(11.4)			
6.0 to 9.9 years	1,115	834 (74.8)	5.4 (6.3)	439.82 (559.04)
(% of Type 1)	(27.8)			
10.0 years or more	2,011	1,596 (79.4)	5.7 (6.3)	464.45 (556.35)
(% of Type 1)	(50.2)			
**Type 2**				
Less than 3.0 years	10,040	5,009 (49.9)	1.6 (2.6)^B^	106.95 (186.54)^B^
(% of Type 2)	(26.7)			
3.0 to 5.9 years	9,004	4,045 (44.9)	1.4 (2.5)	95.31 (189.14)
(% of Type 2)	(23.9)			
6.0 to 9.9 years	10,509	4,974 (47.3)	1.7 (2.9)	116.15 (220.99)
(% of Type 2)	(27.9)			
10.0 years or more	8,072	4,743 (58.8)	2.5 (3.6)	181.48 (286.74)
(% of Type 2)	(21.5)			

A p < 0.001 between duration of diabetes for type 1 diabetes using the Kruskal-Wallis Test

B p < 0.001 between duration of diabetes for type 2 diabetes using the Kruskal-Wallis Test

C p < 0.001 between type 1 and type 2 diabetes for each quartile of follow-up using the Kruskal-Wallis Test
